# Y-chromosomes can constrain adaptive evolution via epistatic interactions with other chromosomes

**DOI:** 10.1186/s12862-018-1327-6

**Published:** 2018-12-27

**Authors:** Ian C. Kutch, Kenneth M. Fedorka

**Affiliations:** Biological Sciences Building, University of Central Florida, 4110 Libra Dr, Orlando, FL 32816 USA

**Keywords:** Y-linked regulatory variation, Epistasis, Evolutionary constraint, *Drosophila melanogaster*, Selection

## Abstract

**Background:**

Variation in the non-coding regions of Y-chromosomes have been shown to influence gene regulation throughout the genome in some systems; a phenomenon termed Y-linked regulatory variation (YRV). This type of sex-specific genetic variance could have important implications for the evolution of male and female traits. If YRV contributes to the additive genetic variation of an autosomally coded trait shared between the sexes (e.g. body size), then selection could facilitate sexually dimorphic evolution via the Y-chromosome. In contrast, if YRV is entirely non-additive (i.e. interacts epistatically with other chromosomes), then Y-chromosomes could constrain trait evolution in both sexes whenever they are selected for the same trait value. The ability for this phenomenon to influence such fundamental evolutionary dynamics remains unexplored.

**Results:**

Here we address the evolutionary contribution of Y-linked variance by selecting for improved male geotaxis in populations possessing multiple Y-chromosomes (i.e. possessed Y-linked additive and/or epistatic variation) or a single Y-chromosome variant (i.e. possessed no Y-linked variation). We found that males from populations possessing Y-linked variation did not significantly respond to selection; however, males from populations with no Y-linked variation did respond. These patterns suggest the presence of a large quantity of Y-linked epistatic variance in the multi-Y population that dramatically slowed its response.

**Conclusions:**

Our results imply that YRV is unlikely to facilitate the evolution of sexually dimorphic traits (at least for the trait examined here), but can interfere with the rate of trait evolution in both males and females. This result could have real biological implications as it suggests that YRV can affect how quickly a population responds to new selective pressures (e.g. invasive species, novel pathogens, or climate change). Considering that YRV influences hundreds of genes and is likely typical of other independently-evolved hemizygous chromosomes, YRV-like phenomena may represent common and significant costs to hemizygous sex determination.

## Background

The capacity for Y-chromosomes to influence the evolution of numerous complex phenotypes has traditionally been viewed as limited, considering that most Y-chromosomes are heterochromatic and comprised of few protein-coding genes [1, 2]. However, Y-chromosomes have recently been shown to influence gene regulation throughout a genome; a phenomenon termed Y-linked regulatory variation (YRV) [3]. This phenomenon provides the potential for hemizygous chromosomes to play a significant role in the evolution of male and female traits. For traits to adaptively evolve via the Y-chromosome, especially sexually dimorphic traits, YRV must induce consistent phenotypic effects within its local gene pool. That is to say, the Y-chromosome must harbor or help to create additive genetic variation. The few studies that have examined Y-linked effects have found no persuasive evidence for additive variance. Instead, these studies (i) found only Y-linked non-additive epistatic variance [4–7], or (ii) were unable to differentiate between additive and epistatic variance due to the examination of Y-linked variation within an isogenic background [8]. Epistasis occurs when the phenotypic contribution of an allele is contingent upon alleles at other loci [9], and can constrain trait evolution by reducing trait heritability and increasing the ruggedness of a trait’s fitness landscape [10]. However, the importance of epistatic variance in evolutionary dynamics has remained controversial [11]; often being dismissed as inconsequential genetic noise [12].

It is important to note that epistasis between the Y and the rest of the genome is different from more common types of epistasis in that it occurs in only one sex. As such, Y-linked epistasis can cause allelic fitness values to differ between males and females, as well as differ among distinctive male genotypes. In the more extreme case of ‘sign’ epistasis (where an allele can produce opposite phenotypic effects depending on the allelic compliment at other loci), allelic fitness values may be entirely reversed, which can theoretically constrain the response to selection [13]. This constraint can influence both sexes given that the X-chromosome and autosomes spend between 33 and 50% of their time in a male genetic background, respectively. If selection promotes entirely different X-chromosome and autosomal alleles when they reside in males, then the population-level response to selection can be dramatically slowed when males and females are selected for the same phenotypic optimum. The degree to which Y-linked epistatic variance will influence male and female evolutionary trajectories will depend on the direction and magnitude of the epistatic effects, as well as the frequency of the epistatically interacting genetic elements in the population [14].

As stated, previous studies in *D. melanogaster* including our own have found no evidence for Y-linked additive variance; instead finding only epistatic variance [4–7]. While these studies provide some information on how Y-linked variation can influence trait evolution, they do not fully address how YRV influences a trait’s response to selection. This is because genetic architecture estimates provide no information about a trait’s fitness landscape; the alteration of which could be substantial if Y-linked variance is sign epistatic and of large magnitude. To more fully assess the evolutionary consequences of YRV, especially if significant sign epistasis is suspected, one should use an approach that incorporates the potential alteration of fitness landscapes, such as laboratory selection studies.

Here, we address whether YRV facilitates or constrains the response to artificial selection for negative geotaxis in *Drosophila melanogaster*; a behavior shown to be influenced by Y-chromosome variation [5]. To this end, we created replicate populations with either multiple Y-chromosomes (constructed from 25 isofemale lines derived from the same wild population; hereafter referred to as Y25 populations) or a single Y-chromosome variant (hereafter referred to as Y1 populations). The Y-chromosomes used in this study have been previously shown to differentially influence immune gene expression when placed in an isogenic background, indicating that they are genetically variable [15]. Furthermore, they have been shown to act epistatically when placed in variable genetic backgrounds [7].

To generate evolutionary change, moderate population-level selection (β = 0.325) for improved negative geotaxis was enacted over 15 generations. If the Y-chromosome contributes significantly to additive variation, then Y25 populations should respond more quickly to selection compared with the Y1 populations. This result would suggest that Y’s have the potential to facilitate the evolution of sexually dimorphic geotactic behavior. However, if Y-chromosomes mostly contribute to epistatic variance, and this variance is of large magnitude, then YRV populations could respond more slowly due to a reduction in trait heritability and/or an increased ruggedness to the trait’s fitness landscape. Regardless of which treatment responds more readily to selection, any significant difference detected between the Y1 and Y25 treatments would suggest that YRV is an important aspect of male genetic architecture. However, a slow responding Y25 population would suggest that epistasis can play a profound role in how traits respond to selection when X-chromosome and autosomal alleles are in a male genetic background.

## Results

**Fig. 1:**
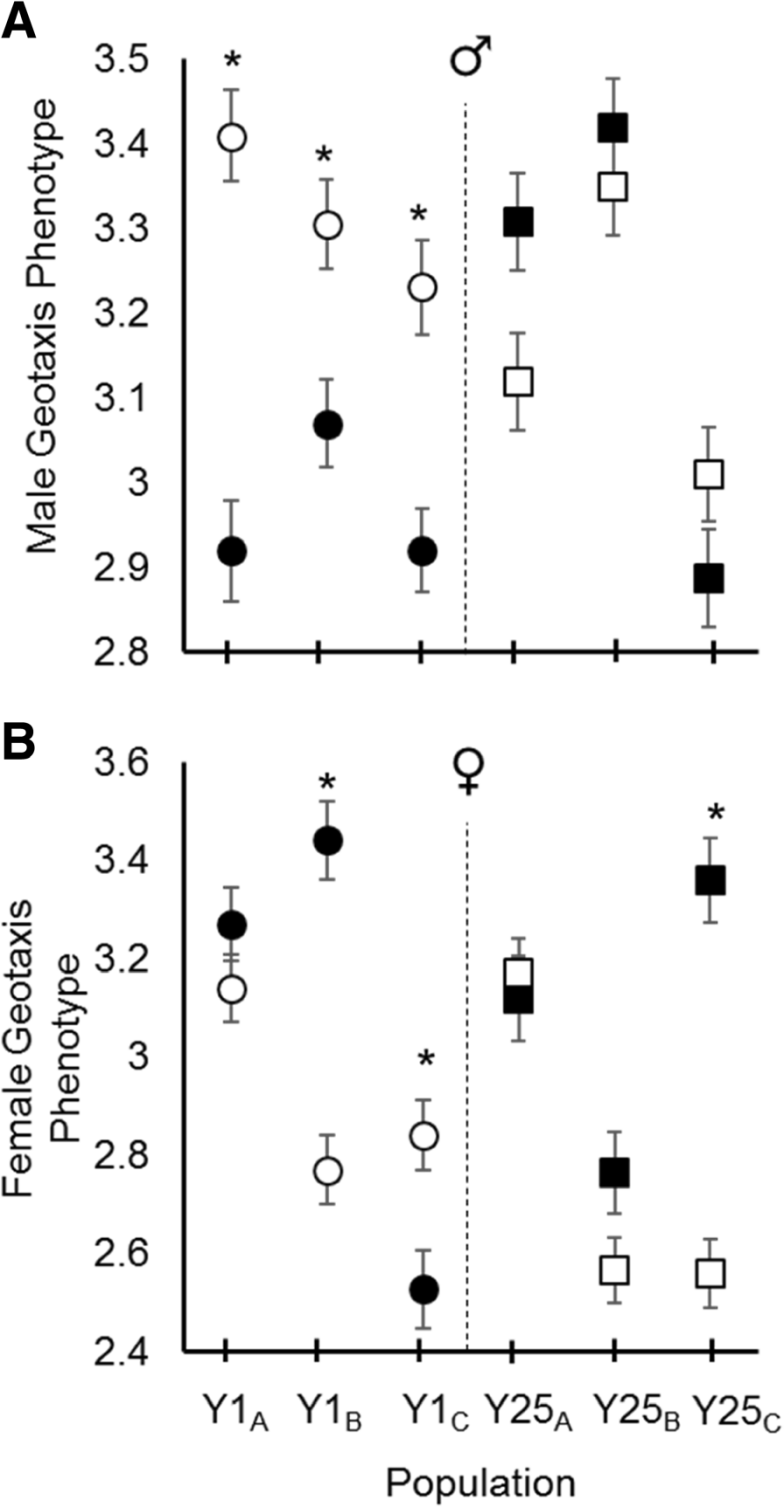
Response to selection. **a** Males from the Y1 replicate populations (circles) responded to selection by increasing negative geotaxis, while males from the Y25 replicate populations (squares) showed no significant increase. Filled symbols represent geotaxis score prior to selection and empty symbols represent geotaxis after selection. **b** Females from the Y1 and Y25 replicate populations showed variation in their response to male selection on the genome. Significant differences between generation 1 and 16 within a population are marked by an asterisk and based on t-tests adjusted for multiple comparisons (k = 6). Error bars represent standard errors. See methods for LS means calculation

**Fig. 2:**
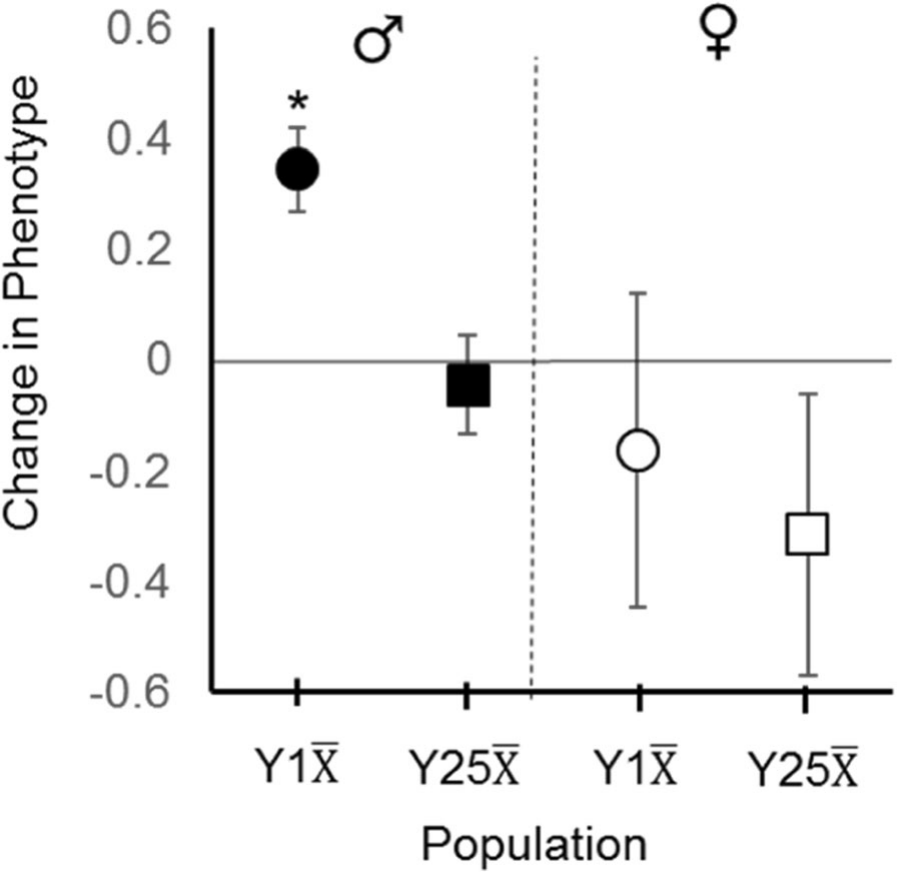
Overall change in geotaxis score after 15 generations of selection. Y1 male geotaxis score (filled circle) increased significantly after selection, while Y25 male geotaxis score (filled square) did not. No overall change in Y1 female (open circle) or Y25 female (open square) was detected. Sex-specific significant differences between Y1 and Y25 geotaxis scores are marked by an asterisk and based on t-tests adjusted for multiple comparisons (k = 2). Data points represent the grand means of each sex-specific treatment (*n* = 3) and error bars represent standard errors. See methods for LS grand means calculation

Prior to selection, replicate populations (A, B, and C) within the Y1 and Y25 treatments differed in their initial geotaxis score for both males (Fig. 1a) and females (Fig. 1b). This was most likely due to chance differences in allele frequencies between replicate populations at the start of the experiment. Overall, the Y1 treatment responded positively to selection, with each replicate population significantly increasing in negative geotaxis from generation 1 to generation 16 (Fig. 1a circles; Table 1). In contrast, the Y25 treatment exhibited no significant change in phenotype in any replicate population (Fig. 1a squares; Table 1). When the change in phenotype in both treatments was examined, the Y1 change in the male grand mean was significantly larger than the Y25 change in the grand mean (Fig. 2; Table 1). These data suggest that the males in populations with multiple Y-chromosomes responded more slowly to selection than the males in populations with a single Y-chromosome variant.

**Table 1:** Statistical assessment of geotaxis phenotypic differences before and after selection for each replicate population, as well as between the Y1 and Y25 grand means

Population	t-test	df	P
Male			
Y1A	5.99	1464	**0.0001**
Y1B	3.10	1547	**0.0020**
Y1C	4.13	1441	**0.0001**
Y25A	2.21	1475	0.0271
Y25B	0.81	1458	0.4177
Y25C	1.50	1541	0.1341
Grand Mean	3.31	4	**0.0296**
Female			
Y1A	1.22	863	0.2215
Y1B	6.14	853	**0.0001**
Y1C	2.88	843	**0.0041**
Y25A	0.46	876	0.6463
Y25B	1.83	963	0.068
Y25C	7.09	891	**0.0001**
Grand Mean	0.40	4	0.7069

Bolded *P*-values are significant after Bonferroni correction

Although selection was not enacted directly on females, selection was enacted on a shared genome. This allowed the consequences of male genomic selection to be assessed in a female background. Females from the Y1 treatment did not exhibit a unified increase in geotaxis like their male counterparts. Instead, Y1 females exhibited all three possible outcomes: a significant increase, decrease, and no change at all (Fig. 1b). With regard to Y25, two populations exhibited no phenotypic change while the third exhibited a significant decrease in geotaxis. When the change in the grand mean phenotype in both treatments was examined, no overall difference was detected (Fig. 2). In sum, these data suggest that male phenotypes behaved consistently and in accordance with epistasis confounding the response to selection. However, female phenotypes were inconsistent at the level of the replicate population, suggesting that YRV may induce unpredictable phenotypic consequences when a male selected genome is placed in a female background.

## Discussion

YRV is a recently discovered phenomenon [3] that has the potential to influence male and female trait evolution, but whose evolutionary consequences have been underexplored. YRV could facilitate sexually dimorphic evolution if it were additive, but constrain evolutionary rates if it were epistatic and of large magnitude. Here, we assessed YRV’s role in geotaxis evolution by selecting for increased negative geotaxis in populations that contained multiple Y-chromosomes variants (i.e. Y25 treatment that possessed YRV) and a single Y-chromosome variant (i.e. Y1 treatment that possessed no YRV). After 15 generations of selection, we show that males of the Y1 populations responded to selection, while males of the Y25 populations did not respond (Fig. 2). This pattern is expected if Y-linked variation constrained adaptive evolution in males. Thus, these data promote the paradigm that YRV is an important aspect of male genetic architecture. Moreover, these data suggest that epistasis can play a profound role in how traits respond to selection; a result that is contrary to some previously published views [12].

The observation that the Y25 males did not respond at all to selection is somewhat curious. Most likely, these males are responding to selection, but responded at a rate slower than we could detect with our experimental design. Still, the reduction in the response rate relative to Y1 appears to be large, leaving one to wonder how autosomal selection can effectively overcome the constraining effects of YRV in order to shape male phenotypes. It is important to note that not all traits are influenced by YRV [3]. Those that are YRV-sensitive likely vary in (1) YRV’s contribution to a trait’s total genetic variance, and (2) the nature of the Y-linked variance (additive versus epistatic). For male geotaxis, it seems that YRV is largely epistatic and contributes greatly to the total genetic variance. Under natural conditions, the extent to which YRV would actually constrain geotactic selection (or selection on any other trait) will ultimately depend on the frequencies of alleles at the interacting genes; including Y-chromosome frequencies [14].

**Fig. 3:**
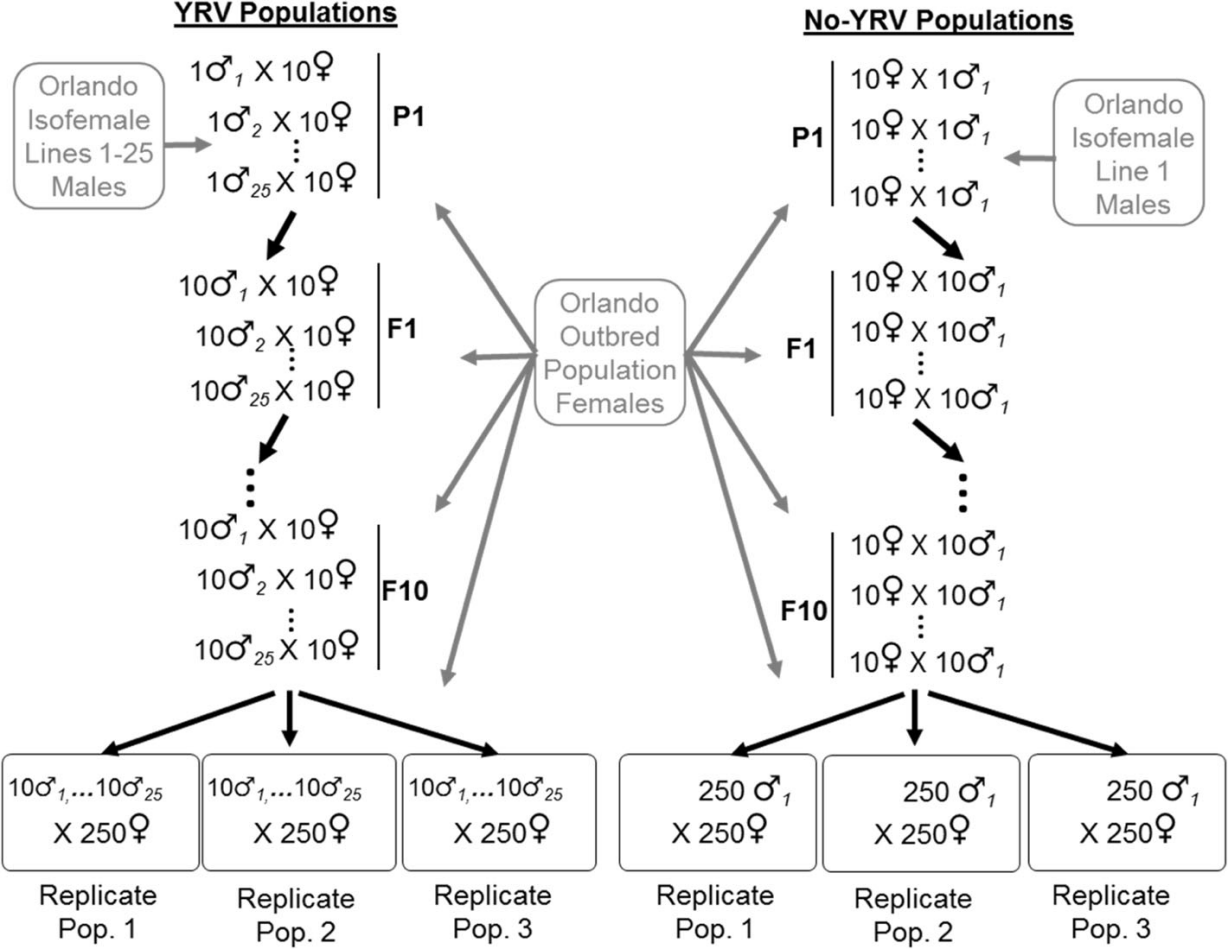
Creation of experimental populations. In the YRV parental generation (P1), 1 male from each isofemale line (1–25) was mated to 10 virgin females from the outbred population. In each subsequent filial generation (F1 - F10), 10 males from the previous line cross were mated to 10 virgin females from the outbred population. By the 10th filial generation, all lines were expected to be 99.9% similar, with the exception of the Y-chromosome. To establish each replicate population, 10 males from each line (*n* = 250 total) were placed in a large cage with 250 virgin females from the outbred population. The same approach was taken with the no-YRV population; however, all males shared the same Y-chromosome. YRV and No-YRV populations were created in parallel. This design maintained similar genetic variation between YRV and no-YRV populations through ample gene flow with outbred base population. All Y-chromosomes started at equal frequencies in the YRV population

In contrast to males, the change in female phenotype among a treatment’s replicate populations was not consistent (Fig. 1b). While the Y1A, Y1B, and Y1C males all showed an increase in negative geotaxis, their female counterparts exhibited all three possible outcomes. Similarly, males of the Y25 replicate populations all showed no phenotypic change, while Y25C females exhibited a large decrease in negative geotaxis. These differences are likely due to (1) the Y-chromosome by autosome epistatic interaction, and (2) differences among the replicate populations in allele frequencies. With regard to Y-chromosome by autosome epistasis, when a male selected genome within a given population is placed into a female background without the interacting Y, the female phenotype could exhibit striking differences from the male phenotype. If sign epistasis is involved, then alleles promoting increased negative geotaxis in males would produce decreased negative geotaxis in females (e.g. Y1B male versus Y1B female). With regard to allelic frequency differences (including Y-chromosomes) between the populations, such differences may cause populations to occupy different localities on similar fitness landscapes or (due to epistasis) cause the landscapes to be different and very rugged [16–18]. If true, then selection could facilitate different paths through the different landscapes; potentially getting marooned on different fitness peaks [19]. This would result in dissimilar genetic composition and phenotypes between the populations at the end of the experiment (e.g. Y1A versus Y1B versus Y1C females). Allelic differences are suggested by the dissimilarities in geotaxis score among the replicate populations at the start of the experiment (Fig. 1), which could have arisen from founder effects when the single treatment population was divided into three (Fig. 3). In short, the male phenotypic pattern is what we expect if YRV is strongly epistatic and of large magnitude, while the female phenotypic pattern could be expected from both Y-linked epistasis and differences in allele frequencies across populations.

It is important to note that Y-linked additive variance may still exist for geotaxis or other traits in *D. melanogaster*, as our study was ultimately limited by our focus on geotaxis, our initial sampling from wild populations, and our experimental design. However, previous work suggests that epistatic variance is the norm [4–7]. Thus, the results reported here may represent typical selection responses for most *D. melanogaster* traits influenced by YRV. If the frequency of the epistatically interacting Y-chromosome variants are minimized by genetic drift, then additive variation could be released [20]. This would then allow selection to adaptively shape dimorphic phenotypes via the Y-chromosome. But given the lack of evidence of Y-linked additive variance cited above, this may be an uncommon occurrence. It’s also important to note that the constraining effect of YRV on male adaptive evolution could constrain female adaptive evolution if selection were sexually monomorphic. In other words, the epistatic interaction between the Y-chromosome and autosomes cause selection to be inefficient when the autosomes are in a male background, slowing down the rate at which the sexes achieve a new, shared phenotypic optimum. This result has real biological implications, as it can impact how quickly a population responds to a new selective pressure, such as an invasive species, a novel virulent pathogen, or climate change.

In this study, our goal was to compare populations that possessed Y-linked variation (Y25 populations) to those that did not possess Y-linked variation (Y1 populations). To this end, our experimental design was appropriate. One potential caveat with this design, however, is the use of the same Y-chromosome variant to establish our Y1 replicate populations. Under our stated goal, this design attribute should not be an issue, as different Y-chromosomes across the Y1 replicate populations would only adjust the population’s phenotypic mean, not its genetic variance (which is ultimately the fuel necessary for evolutionary change). The caveat arises only if Y-chromosomes can act a capacitors of additive genetic variation; that is to say, variable switches that turn on or turn off cryptic genetic variance elsewhere in the genome. If true, our results could be due to inadvertently selecting a Y-chromosome for the Y1 populations that released cryptic additive genetic variance in geotaxis. Although there is some evidence for the existence of genetic capacitors in nature [21, 22], the extent to which such capacitors exist and impact evolution is highly controversial [23]. Furthermore, there is no evidence that Y-chromosomes can act in such a capacity. Thus, we view this possibility as intriguing but remote.

## Conclusions

Our data suggest that Y-linked variance in *D. melanogaster* can retard the rate of adaptive evolution in males and possibly females. Furthermore, the data suggest that epistatic variance can play a significant role in evolutionary dynamics. Given that the Y’s influence on genome regulation has been seen in multiple species [24], is associated with its heterochromatic landscape [3, 25], and independently evolved Y-chromosomes tend to be comprised largely of heterochromatin [2], the limitation on evolutionary rates enacted by YRV-like phenomena may represent a common and substantial cost to hemizygous sex determination. As such, a deeper examination of this phenomenon beyond what is presented here is warranted.

## Methods

### Experimental populations

All flies used in this study were collected in the fall of 2011 from a single location in Orlando Florida. Collection and maintenance of flies complied with all known guidelines. Isofemale lines were established by isolating offspring from the originally collected wild females and enacting single-sibling matings over 10 generations to create near-isogenic genotypes. Isofemale lines were then divided and half of each line was combined to form an outbreeding population, which was maintained with a large number of breeding adults each generation (approximately 1000) for 4 years prior to the experiment. The degree to which the isofemale lines were isogenic prior to the creation of the outbred population is unimportant, as extensive unidirectional migration from the outbred population to each isofemale line was later enacted to create the experimental populations (see below; Fig. 1). What is important is that each of these isofemale lines contains a single and potentially different Y-chromosome.

To create the experimental Y25 populations, males from the 25 isofemale lines were backcrossed to virgin females from the outbred population for 10 generations (Fig. 3). This created 25 Y-lines that were genetically similar to the outbred population, but each contained a potentially unique Y-chromosome. In the following generation, three replicate Y25 populations were created by taking 30 males from each line and distributing them equally across three population cages. To each cage, 250 virgin females from the outbred population were then added. The Y1 populations were created in the same manner, except that a single, randomly chosen isofemale line was used instead of all 25 isofemale lines (Fig. 3). Both Y1 and Y25 populations were constructed simultaneously and, based on the experimental design, were assumed to be comprised of similar genetic variation with similar allele frequencies. During the selection experiment, replicate populations were maintained on a 2-week generation cycle on a sugar, cornmeal, yeast, and agar *Drosophila* media. All flies were kept in a Percival incubator at 27 °C on a 12 h:12 h light dark cycle.

### Artificial selection

**Fig. 4:**
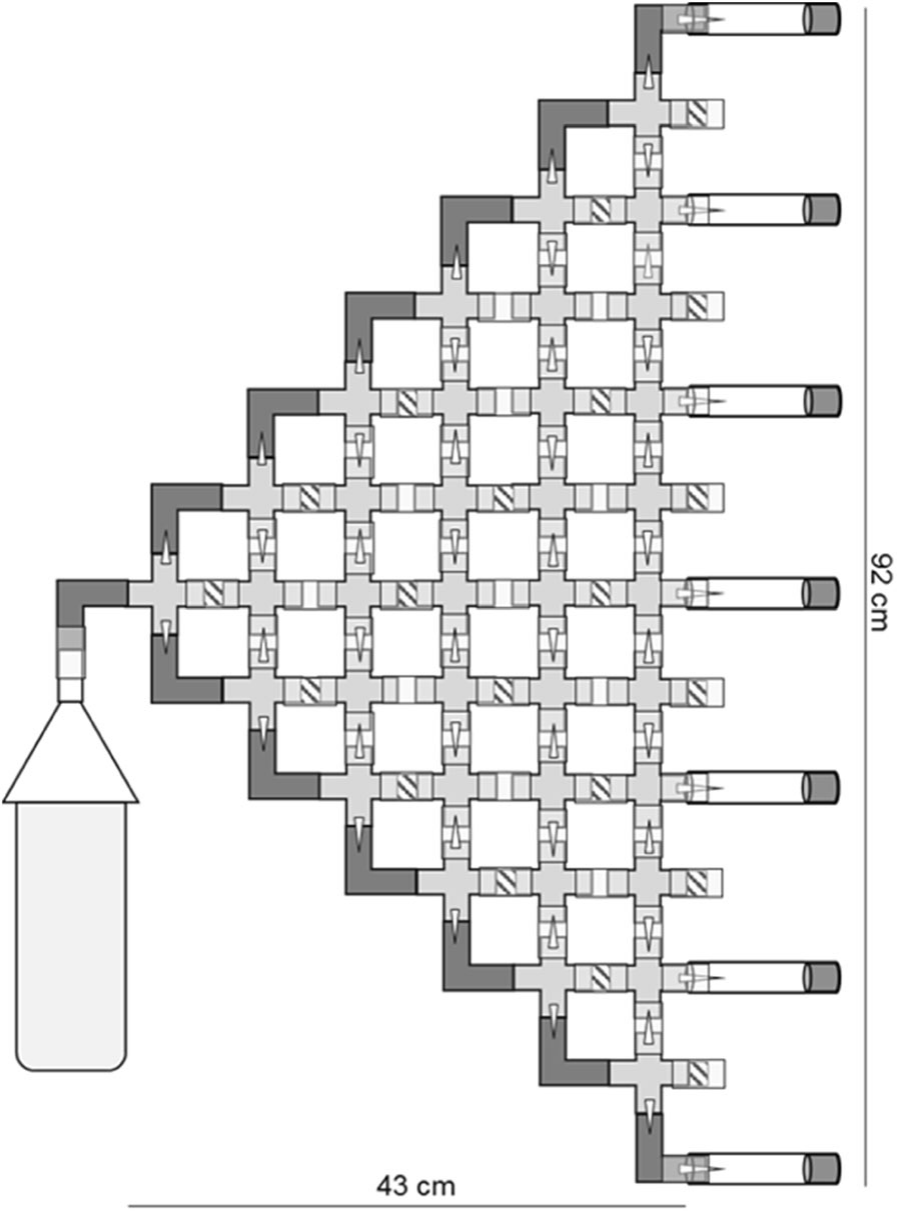
Geotaxis maze. Seven mazes were constructed in identical fashion, using 36 PVC cross junctions, 13 PVC elbow joints, 49 1 ml pipette tips embedded in hot glue, and numerous pieces of 0.5 in. diameter vinyl tubing to connect the cross junctions. Hot glue was also used to block unwanted horizontal movement (designated by diagonal lines) and pipette tips created one-way passage into an adjacent cross junction. Vials filled with food were placed at the terminal exits to attract flies. This maze provided flies with a total of 6 up or down choices

To artificially select for improved negative geotaxis, seven identical geotaxis mazes were constructed using 4-way PVC cross junctions interconnected with 0.5 in. diameter vinyl tubing (Fig. 4). Within each junction, the vertical exits were fitted with modified pipette tips imbedded in hot glue, providing flies with a one-way choice up or down. The lateral exit was sealed with hot glue to prevent horizontal movement. Each maze was comprised of 36 cross junctions to create 7 potential exits of varying height that were caped with vials of food. The maze entrance was composed of a 1 l plastic bottle that allowed flies to gradually move into the maze of their own accord.

In each generation, 300 virgin male flies from each population were placed at the entrance to the geotaxis maze and allowed to disperse (*n* = 1800 total flies per generation). Mazes were systematically assigned to each population such that each population experienced all seven mazes the same number of times. Twenty-four hours after flies were placed into the maze, they were scored based on their exit vial, which represented the number of up or down choices made. The average phenotypic score (0–6) for each population was then standardized to a mean of zero and unit variance. In order to determine which flies founded the next generation, regression analysis was used. In short, all flies were initially assigned a fitness score of 0 (non-breeder). Starting at the top of the phenotypic distribution (i.e. most negatively geotactic), each fly was sequentially assigned a fitness value of one (breeder) until a slope (i.e. selection gradient) of 0.65 was reached. Given that selection was only imposed on male flies, the population level selection gradient was 0.325; a strength of selection that allowed for the observation of phenotypic change in a reasonable time but not strong enough to quickly fix Y’s in our populations. Although this gradient is weak compared with other geotaxis selection studies [26–28], it is typical of selection gradients in nature for a wide range of traits [29]. Each generation, selected male flies were allowed to mate with unselected virgin females and lay eggs for 48 h. Adults were then culled from media bottles. The next generation was collected as virgins and aged 4 days before being again selected. Mazes were rinsed with ethanol and water between geotaxis assays and then blown dry using pressurized air circulated through the maze for 30 min. Laboratory selection was enacted for 15 generations.

### Selection response

To assess how populations responded to artificial selection, robust measures of negative geotaxis for both males and females were conducted at the start (generation one, prior to selection) and end (generation 16) of the experiment. To estimate the male phenotype in generation one, approximately 300 naive males from each experimental population were randomly assigned to a maze and allowed to traverse the maze from 9 am to 8 pm. Each population was simultaneously assessed over three consecutive days and each day new males were utilized, resulting in a total of 5226 assayed males. The same was done for the female phenotypic estimates, except that approximately 150 females from each population were assayed at night from 9 pm to 8 am, resulting in a total of 2003 assayed females. Between male and female maze runs, the mazes were cleaned between assays as described above. At generation 16, robust phenotypic measures were again conducted in a similar manner, except that approximately 100 males and 100 females per population were used each day over seven consecutive days, resulting in 3712 total males and 3298 total females. For these estimates, mazes were systematically assigned so that each population used every maze only once. In generation 1 and 16 a combined 14,239 male and female flies were assessed.

### Statistical analysis

To assess the effect of our treatment, we first generated sex-specific least-squares means for each replicate population’s geotaxis phenotype before and after selection, resulting in the generation of 24 LS means (three replicate populations per treatment per sex at generation 1 and 16). For each sex and generation, we employed a standard least squares model with maze exit score as the response variable, and population, maze, and trial day as dependent variables. The resulting LS means and variances were then used to assess the sex-specific statistical differences in phenotype between generation 1 and generation 16 within each replicate population using t-tests corrected for multiple comparisons (k = 6). To determine if the Y1 treatment responded differently to selection than the Y25 treatment, we calculated the overall change in sex-specific phenotype (LS mean post selection – LS mean pre selection) for each replicate population. These means were then used to determine if differences existed between Y1 and Y25 in their grand mean using a t-test. Males and females were analyzed separately considering that they were assayed at different times of the day (day and night, respectively), which influences the geotaxis phenotype (ICK personal observation). All statistical analysis were performed in JMP v.12.

## Abbreviations

LS: Least squares
Y1: Moniker for populations that were founded by males derived from one of the Y-lines established from a wild caught female
Y25: Moniker for populations that were founded by males derived from 25 Y-lines independently established from wild caught females
YRV: Y-linked regulatory variation
